# Multi-Wavelength Photobiomodulation Therapy Combined with Static Magnetic Field on Long-Term Pulmonary Complication after COVID-19: A Case Report

**DOI:** 10.3390/life11111124

**Published:** 2021-10-22

**Authors:** Shaiane Silva Tomazoni, Douglas Scott Johnson, Ernesto Cesar Pinto Leal-Junior

**Affiliations:** 1 Physiotherapy Research Group, Department of Global Public Health and Primary Care, University of Bergen, 5020 Bergen, Norway; ernesto.leal.junior@uninove.br; 2 Multi Radiance Medical, Solon, OH 44139, USA; djohnson@multiradiance.com; 3 Laboratory of Phototherapy and Innovative Technologies in Health (LaPIT), Post-Graduate Program in Rehabilitation Sciences, Nove de Julho University, São Paulo 01525-000, Brazil; 4 ELJ Consultancy, Avenida Chibarás, São Paulo 04076-000, Brazil

**Keywords:** low-level laser therapy, COVID-19, rehabilitation, pulmonary complication, case reports

## Abstract

Introduction: Photobiomodulation therapy, alone (PBMT) or combined with a static magnetic field (PBMT-sMF), has been demonstrated to be effective in the regeneration of tissues, modulation of inflammatory processes, and improvement in functional capacity. However, the effects of PBMT-sMF on the pulmonary system and COVID-19 patients remain scarce. Therefore, in this case report, we demonstrated the use of PBMT-sMF for peripheral oxygen saturation, pulmonary function, massive lung damage, and fibrosis as a pulmonary complication after COVID-19. Case report: A 53-year-old Mexican man who presented with decreased peripheral oxygen saturation, massive lung damage, and fibrosis after COVID-19 received PBMT-sMF treatment once a day for 45 days. The treatment was irradiated at six sites in the lower thorax and upper abdominal cavity and two sites in the neck area. We observed that the patient was able to leave the oxygen support during the treatment, and increase his peripheral oxygen saturation. In addition, the patient showed improvements in pulmonary severity scores and radiological findings. Finally, the patient presented with normal respiratory mechanics parameters in the medium-term, indicating total pulmonary recovery. Conclusions: The use of PBMT-sMF may potentially lead to safe treatment of and recovery from pulmonary complications after COVID-19, with regard to the structural and functional aspects.

## 1. Introduction

Severe acute respiratory syndrome coronavirus (SARS-CoV-2) is the causative agent of the novel coronavirus disease (COVID-19) [1]. The clinical features triggered by SARS-CoV-2 infection may differ in each patient. Patients may be asymptomatic or, in some cases, patients may develop life-threatening sepsis [1,2]. In its early stages, SARS-CoV-2 targets pneumocytes and nasal and bronchial epithelial cells, and it infects and kills T lymphocyte cells [2,3]. In later stages, there is an enhanced inflammatory response when the virus infects pulmonary capillary endothelial cells [4]. In addition, SARS-CoV-2 promotes endothelial barrier disruption, dysfunctional alveolar–capillary oxygen transmission, and impaired oxygen diffusion capacity [2]. These pulmonary characteristic features caused by SARS-CoV-2 may persist as pulmonary complications after treating COVID-19. There is evidence that a considerable proportion of patients have a pulmonary diffusion abnormality 6 months after COVID-19 symptoms onset [5].

Multi-wavelength photobiomodulation therapy (PBMT) combined with static magnetic field (PBMT-sMF) may be a non-pharmacological alternative intervention for the treatment of persistent pulmonary complications after COVID-19. PBMT is a light therapy that uses non-ionizing light sources such as low-level lasers, light emitting diodes, and broadband light from the visible to the infrared spectra to promote pain relief, regenerate tissues, and modulate inflammatory processes [6,7]. In the respiratory system, PBMT has been able to modulate pulmonary inflammation and relieve bronchial hyperresponsiveness in experimental models [8,9,10], in addition to improving the functional capacity of patients with chronic obstructive pulmonary disease [11]. In recent years, PBMT has been used in combination with a static magnetic field to generate greater electron transfer and, consequently, better effects on cell metabolism [12]. The positive effects of PBMT-sMF have been shown to be similar to those of PBMT alone, such as ergogenic effects, inflammatory process modulation, and pain relief [13,14,15]. However, currently, there is insufficient evidence regarding the effects of PBMT-sMF on the respiratory system. In addition, there is a lack of evidence on the effects of PBMT alone or PBMT-sMF on pulmonary complications after COVID-19. Here, we report the use of PBMT-sMF on peripheral oxygen saturation (SpO_2_), pulmonary function, massive lung damage, and fibrosis as a pulmonary complication after COVID-19.

## 2. Case Report

A 53-year-old Mexican man, who was overweight and had a history of serological antibody tests for toxocariasis positive, brucellosis, gout, conjunctivitis, rhinitis, seasonal allergies, sneezing, and nasal congestion, presented with generalized pain, fatigue, intermittent fever (38.5–40 °C), dry cough, and dyspnea. SARS-CoV-2 diagnosis was confirmed by reverse transcription polymerase chain reaction (RT-PCR) using a nasopharyngeal swab. The patient was admitted to the hospital with an SpO_2_ of 74% and oxygen support of 14 L/min. Chest radiography demonstrated massive lung damage and fibrosis caused by severe pneumonia. At the hospital, the patient was treated with ceftriaxone and prednisone, along with enoxaparin injections and oral aspirin. After 10 days at the hospital, the patient improved, and he was discharged from the hospital. However, the patient still presented with generalized fatigue, and oxygen support (2 L/min) was still required for sleeping and eating. Therefore, ten days after discharge from the hospital, the patient sought complementary treatment with PBMT-sMF to improve his health status.

### 2.1. Pretreatment Clinical Findings

The patient’s response to the PBMT-sMF treatment was evaluated by monitoring SpO_2_ [16,17] from the time of admission to the hospital (baseline), until discharge from the hospital, 10 days after the start of treatment, immediately after the end of the treatment, and 4 months after the end of the treatment with PBMT-sMF. In addition, we evaluated the progression of the imaging findings in the chest X-ray from the first X-ray at baseline, until 10 days after the start of the treatment, and 4 months after the end of the treatment with PBMT-sMF. Finally, to quantify the extent of SARS-CoV-2 infection in the lungs, the severity score was calculated from chest X-rays. The adapted and simplified Radiographic Assessment of Lung Edema (RALE) score was used [18,19]. A score ranging from 0 to 4 was assigned to each lung based on the extent of consolidation or ground glass opacity: 0, no involvement; 1, <25% involvement; 2, 25–50% involvement; 3, 50–75% involvement; and 4, >75% involvement. The final severity score was calculated as the sum of the scores for each lung. The RALE score was calculated at baseline, 10 days after the start of treatment, and 4 months after the end of the treatment with PBMT-sMF.

### 2.2. Intervention

**Figure 1:**
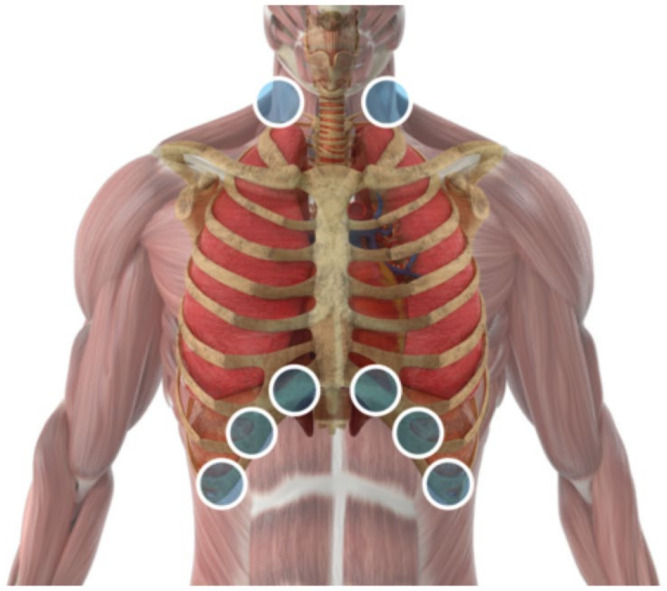
Intervention sites where PBMT-sMF was irradiated. Six sites in the lower thorax/upper abdominal cavity and two sites in the neck area.

Ten days after discharge from the hospital, the patient started the multi-wavelength PBMT-sMF treatment. PBMT-sMF was irradiated using a cordless, portable MR5 LaserShower™ device (Multi-Radiance Medical™, Solon, OH, USA). Six sites in the lower thorax/upper abdominal cavity were irradiated in addition to two sites in the neck area (Figure 1). At each treatment session, the patient received a total of 31.50 J per irradiated site. The treatment time per site was 60 s, yielding 480 s per treatment session. The treatment was performed once daily for 45 days. The dose and irradiation sites were established based on previous studies [11,20]. Table 1 describes the PBMT-sMF parameters.

### 2.3. Post-Treatment Outcomes

After 10 days of PBMT-sMF treatment, the SpO_2_ of the patient increased from 89% to 93% at 2 L/min oxygen. After 40 days of treatment with PBMT-sMF, the patient was able to leave the oxygen support. After 45 days, at the end of treatment with PBMT-sMF, the patient’s SpO_2_ was at 96–98%. Finally, in the last evaluation, 4 months after PBMT-sMF treatment, the patient’s SpO_2_ was at 98% (Table 2).

**Figure 2:**
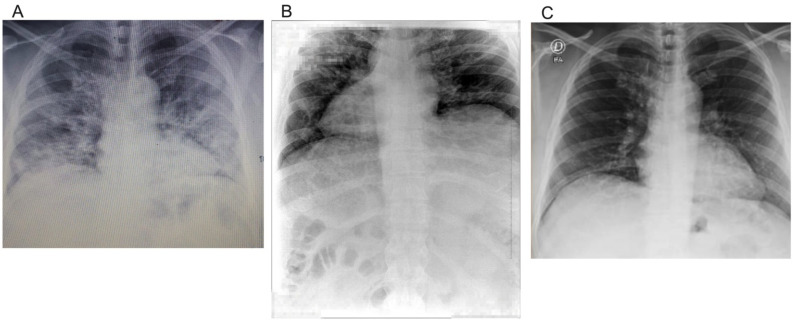
Radiological findings at baseline, 10 days after intervention and 4 months follow-up. Radiological findings at baseline (**A**). At baseline, the extension of consolidation or ground glass opacity was greater than 75% in the right lung, and 50–75% in the left lung. The figure is owned by the patient. Radiological findings at 10 days after starting the PBMT-sMF treatment (**B**). 10 days after starting the PBMT-sMF treatment, the extension of consolidation or ground glass opacity was greater than 75% in both lungs. The figure is owned by the patient. Radiological findings at 4 months follow-up evaluation (**C**). At 4 months follow-up evaluation, there was no consolidation or ground glass opacity in both lungs. The figure is owned by the patient.

Figure 2 shows the extent of SARS-CoV-2 infection in the lungs. In addition, this was the basis for measuring the RALE score. When the patient was admitted to the hospital, his RALE score was 7 (Figure 2A). However, on the second chest radiograph, his RALE score worsened to 8 (Figure 2B). Finally, on the last radiograph, at 4 months follow-up evaluation, his RALE score was 0, indicating total recovery of the lungs (Figure 2C) (Table 2).

**Figure 3:**
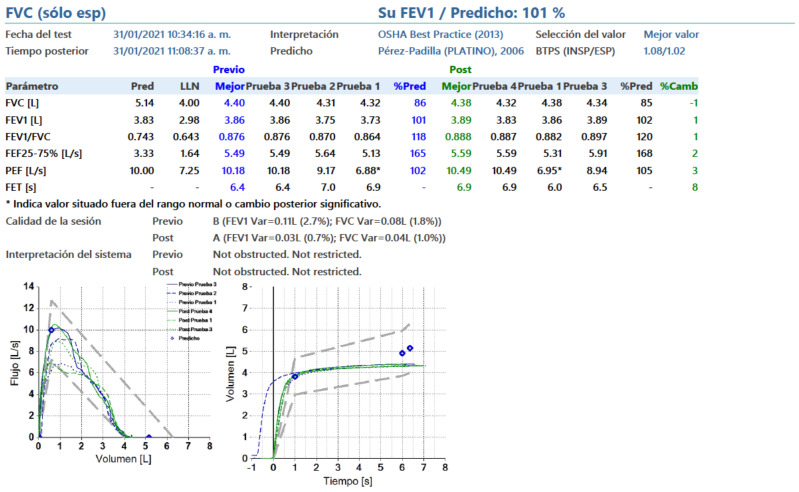
Spirometry–pulmonary function tests at 4 months follow-up evaluation. The respiratory mechanics parameters were found according to their prediction, that is, what is normal in both the pre-and post-bronchodilator. The figure is owned by the patient.

At the 4 months follow-up, spirometry was performed to assess the sequelae after COVID-19. Respiratory mechanics parameters were found according to their prediction, that is, what is normal in both the pre-and post-bronchodilator (Figure 3). The patient did not report any adverse events associated with the use of PBMT-SMF.

## 3. Discussion

This case report showed that the patient treated with PBMT-sMF for 45 days presented an increase in SpO_2_, in addition to improvements in oxygen support requirements, pulmonary severity scores, and radiological findings. In addition, during the 45-day treatment course with PBMT-sMF, the patient was able to leave the oxygen support. At follow-up 4 months after PBMT-sMF treatment, the clinical recovery of the patient was total, which was mainly proven by the pulmonary recovery.

These findings are consistent with the effects previously demonstrated by PBMT alone or by PBMT-sMF, such as the regeneration of tissues and modulation of the inflammatory process in several tissues [6,7] and in the respiratory system [8], as well as decreased pulmonary fibrosis and improvements in pulmonary functional capacity [11,21]. In addition, our findings corroborate a previous case report that demonstrated that PBMT treatment in a patient with severe COVID-19 was beneficial in reducing inflammatory markers and improving respiratory indices and radiological findings [16]. Moreover, another previous case report demonstrated improvements in respiratory indices, oxygen requirements, and radiological findings in a patient with severe COVID-19 treated with PBMT [17]. It is important to highlight that the aforementioned case reports only showed short-term improvements in patients, while our study demonstrated that patients treated with PBMT-sMF for 45 days also had better clinical findings at the long-term evaluation (4 months after the treatment).

Patients with COVID-19 often progress to acute respiratory distress syndrome, with an increased presence of cytokines such as interleukin (IL)-1β and IL-6. The findings observed in the present case report suggest that PBMT-sMF could modulate the inflammatory process, possibly decreasing the levels of these cytokines, as observed in previous experimental studies [22,23]. Another possible mechanism of action is that PBMT-sMF may contribute to the modulation of the immune system by acting on interferons [24]. In addition, PBMT alone or PBMT-sMF can reduce the production of reactive oxygen species and may lead to reduced oxidative stress in this specific condition [8,25]. Finally, PBMT-sMF may have been beneficial in preserving and improving the respiratory muscles of the patient. Therefore, our findings suggest that PBMT-sMF, when applied to the accessory muscle of respiration, helps improve the inflammatory process caused by COVID-19 by modulating inflammatory markers and the immune system, in addition to reducing oxidative stress. The combination of these positive effects of the use of PBMT-sMF contributed to the improvement in tissue oxygenation, pulmonary inflammation, and general clinical condition, which helped in the total recovery of the patient.

Although, currently there are no known adverse effects related to the use of PBMT-sMF, there are some contraindications regarding its use that should be carefully observed, such as irradiation in areas of infection, active carcinoma, and the pelvic and thoracoabdominal region in pregnant women.

One of the strengths of this case report was that we performed a medium-term follow-up of the patient to verify the evolution of the outcomes after the use of PBMT-sMF. In addition, we observed clinically important outcomes for the patient, such as peripheral oxygen saturation and pulmonary changes. However, one of our limitations is that our study was retrospective; thus, we do not have all the outcomes collected at all timepoints. In addition, the patient was diagnosed and treated at the critical time of the COVID-19 outbreak. Thus, the health system was overwhelmed, and access to the hospital and tests were limited. Therefore, the patient had to perform the second chest X-ray in a veterinary clinic, because he was a veterinarian and had access to veterinary facilities, and other laboratory tests could not be performed.

To corroborate our findings, randomized controlled trials with rigorous methodological quality and an adequate sample size are needed to investigate the effects of PBMT-sMF on pulmonary complications after COVID-19 in the short-, medium-, and long-term. In addition, studies on the effects of PBMT-sMF on patients with COVID-19 are needed.

## 4. Conclusions

The use of multi-wavelength PBMT-sMF may potentially lead to the safe treatment of and recovery from long-term pulmonary complications after COVID-19 infection, with regards to the structural and functional aspects.

## Figures and Tables

**Table 1:** Full description of the PBMT-sMF parameters.

	Lower Thorax/ upper Abdominal Cavity	Neck Area
Number of lasers	4	4
Wavelength (nm)	905	905
Frequency (Hz)	250	250
Peak power (W)-each	50	50
Average mean optical output (mW)-each	1.25	1.25
Power density (mW/cm^2^)-each	3.91	3.91
Energy density (J/ cm^2^)-each	0.234	0.234
Dose (J)-each	0.075	0.075
Spot size of laser (cm^2^)-each	0.32	0.32
Number of red LEDs	8	8
Wavelength of red LEDs (nm)	633	633
Frequency (Hz)	2	2
Average optical output (mW)-each	25	25
Power density (mW/cm^2^)-each	29.41	29.41
Energy density (J/ cm^2^)-each	1.765	1.765
Dose (J)-each	1.50	1.50
Spot size of red LED (cm^2^)-each	0.85	0.85
Number of infrared LEDs	8	8
Wavelength of infrared LEDs (nm)	850	850
Frequency (Hz)	250	250
Average optical output (mW)-each	40	40
Power density (mW/cm^2^)-each	71.23	71.23
Energy density (J/ cm^2^)-each	4.286	4.286
Dose (J)-each	2.40	2.40
Spot size of infrared LED (cm^2^)-each	0.56	0.56
Magnetic field (mT)	110	110
Irradiation time per site (sec)	60	60
Total dose per site (J)	31.50	31.50
Number of irradiated sites	6	1 (bilaterally)
Total dose delivered to the muscle group (J)	189.00	31.50 (bilaterally)
Aperture of device (cm^2^)	33	33
Application mode	Cluster probe held stationary in skin contact with a 90-degree angle and slight pressure	Cluster probe held stationary in skin contact with a 90-degree angle and slight pressure

LED: light-emitting diode.

**Table 2:** Post-treatment outcomes.

Variables.	Baseline	Discharge from Hospital	10 Days of PBMT-sMF	After End of Treatment	4 Months Follow-Up
SpO_2_ (%)	74	89	93	96-98	98
RALE	7	--	8	--	0

SpO_2_ = peripheral oxygen saturation; RALE = radiographic assessment of lung edema; PBMT-sMF: photobiomodulation therapy combined with static magnetic field.

## Data Availability

All datasets generated for this study are included in the article.
